# Genetic and pharmacological targeting of A2a receptor improves function of anti-mesothelin CAR T cells

**DOI:** 10.1186/s13046-020-01546-6

**Published:** 2020-03-10

**Authors:** Elham Masoumi, Leila Jafarzadeh, Hamid Reza Mirzaei, Khadijeh Alishah, Keyvan Fallah-Mehrjardi, Hosein Rostamian, Mohammad Khakpoor-Koosheh, Reza Meshkani, Farshid Noorbakhsh, Jamshid Hadjati

**Affiliations:** 1 Department of Medical Immunology, School of Medicine, Tehran University of Medical Sciences, Tehran, Iran; 2 Department of Biotechnology, Faculty of Science, University of Tehran, Tehran, Iran; 3 Department of Clinical Biochemistry, School of Medicine, Tehran University of Medical Sciences, Tehran, Iran

**Keywords:** Mesothelin, Chimeric antigen receptor, Adenosine 2a-receptor, Genetic targeting, Pharmacological targeting, Tumor microenvironment

## Abstract

**Background:**

CAR T cell-based therapies have shown promising results in hematological malignancies. Results of CAR T cell projects in solid tumors have been less impressive, and factors including lack of targetable antigens and immunosuppressive tumor microenvironment (TME) have been suggested as culprits. Adenosine, a metabolite which is highly produced in TME, is known to mediate the suppression of anti-tumor T cell responses via binding and signaling through adenosine 2a receptor (A2aR).

**Methods:**

In this study, the expression of A2aR and the effects of its activation on the function of fully human anti-mesothelin CAR T cells (MSLN-CAR T), were analyzed. Afterwards, the molecular and pharmacological means to overcome the inhibitory effects of A2aR signaling on CAR T cell function were used. This was performed by targeting A2aR expression in MSLN-CAR T cells using various anti-A2aR shRNA sequences embedded in the CAR vector and an A2aR pharmacological antagonist, SCH-58261. Statistical analyses were performed Prism 7 software.

**Results:**

Our experiments showed significant A2aR upregulation on T cells during the CAR T cell production procedure (cell activation and transduction). Activation of adenosine signaling using adenosine analog led to the suppression of all major anti-tumor functions in MSLN-CAR T cells. Interestingly, CAR T cells that carried the anti-A2aR shRNA sequences were resistant to the inhibitory effects of adenosine signaling. Pharmacological inhibition of A2aR reversed the reduction in CAR T cell proliferation and cytokine response caused by the adenosine analog; however, it failed to rescue the cytotoxic function of the cells.

**Conclusion:**

Altogether, our results indicate that mitigating A2aR signaling by genetic targeting of the receptor might be a promising approach in improving CAR T cell function in an unreceptive microenvironment and could potentially improve the outcome of treatment in clinical settings.

## Background

CAR T cells are engineered to express antibody-derived single-chain variable fragments (scFv) against tumor antigens combined with appropriate intracellular signaling domains. CAR T cell-based therapies have shown promising results in the treatment of hematological malignancies including CD19 or CD22 positive B-cell acute lymphoblastic leukemia (ALL). However, despite the abundant research, treatment of solid tumors by CAR T cells has been less successful. Various factors have been suggested to contribute to such a lack of efficacy, including the scarcity of targetable tumor associated antigens and hostile TME, which might act as a key impediment for T cell function [1].

A common feature of solid tumors’ micro-environment is low oxygen pressure [2]. To adapt to the hypoxic conditions, cells initiate several survival mechanisms including the activation of HIF-1α pathway which has a key role in tissue vascularization in hypoxic situations [3, 4]. HIF-1α induces two ectonucleotidases, CD73 and CD39, on the surface of both tumor and immune cells. These molecules convert adenosine triphosphate (ATP) to adenosine in two separate steps [5]. Adenosine is known to exert immunosuppressive effects within the TME [3]. Four different receptors have been heretofore discovered for adenosine, A1, A2a, A2b, and A3 receptors [6]. A2aR which is expressed at the surface of activated T cells, binds to adenosine with high affinity and leads to enhanced production of intracellular cyclic AMP (cAMP). Elevated levels of cAMP can attenuate the anti-tumor T cell responses [4, 7, 8].

In the present study, we designed a fully human second generation anti mesothelin-CAR T cell (MSLN-CAR T). To mitigate the effects of TME on CAR T cells, we combined CAR expression with the expression of shRNA sequences against the A2aR gene (A2aR.KD.MSLN-CAR T cell). Experiments were performed to evaluate the function of CAR T cells in the absence and/or presence of A2aR knockdown (KD). Different aspects of the T cell function including proliferative response, cytotoxic activity and cytokine production were evaluated. To simulate TME, T cell functional analyses were also performed in the presence of an A2aR specific agonist. Moreover, to compare the effects of A2aR knockdown with pharmacological inhibition, functions of CAR T cells were evaluated in the presence of a specific A2aR inhibitor.

## Materials and methods

### Cell lines

Hela, Skov3 and Ovcar3 as mesothelin-expressing cell lines, the Nalm-6 as a mesothelin negative cell line, and HEK293T as packaging cell line were all purchased from the Iranian Biological Resource Center (IBRC). Before the experiments, mesothelin expression levels were analyzed for Skov3, Ovcar3, Nalm-6 and HeLa cells by flow cytometry using PE-conjugated anti-human mesothelin antibody (R&D Systems, Minneapolis, MN, USA). HeLa cells showed approximately 55% mesothelin expression and were selected as the cell line with the highest level of target antigen expression (data not shown). HEK293T and Hela cells were cultured in Modified Eagle Medium (DMEM) (Gibco Laboratories, Grand Island, NY) and the other cells in RoswellPark Memorial Institute (RPMI) 1640 (Gibco Laboratories) both supplemented with 10% fetal bovine serum (FBS) (Gibco Laboratories), 1% penicillin/streptomycin (PAN Biotech, Aidenbach, Germany), and 2 Mm L-glutamine and incubated at 37 °C in 5% CO2. The absence of mycoplasma was confirmed for all cell lines by PCR.

### CAR synthetics

The fully human CAR sequence is composed of a kozak consensus sequence, a human CD8 signal peptide (SP), a fully human anti mesothelin scFv, CD8a hinge domain, the transmembrane (TM) region of the human 4-1BB molecule, and an intracellular signaling domain containing both 4-1BB and CD3ζ domains (MSLN-CAR). Mesothelin-specific scFv fragment was originated from P4-scFv [9] and other fragments of CAR construct were described previously [10]. To target A2aR, three shRNA-coding sequences were designed to target three different segments of the A2aR gene. shRNA-coding sequences contained 5′-Flank-Sense-Loop-Antisense-3’Flank segments, and were inserted after CD3ζ domain sequence in CAR constructs (A2aR.KD1.MSLN-CAR, A2aR.KD2.MSLN-CAR, and A2aR.KD3.MSLN-CAR). The sequences of shRNAs are shown in Table 1. The anti-mesothelin CAR genes were cloned into the pCDH-CMV-MCS-EF1α-GreenPuro lentivector backbone (third generation) for generating lentiviral particles.

**Table 1:** shRNA sequences for targeting A2aR transcripts

Name	Sense sequence	Anti-sense sequence	Target gene
shRNA1 (KD1)	CTCGGTGTACATCACGGTGGAG	CTCCACCGTGATGTACACCGAG	A2a receptor (ADORA2A)- NM_000675.5
shRNA2 (KD2)	GAACTACATGGTGTACTTCAAC	GTTGAAGTACACCATGTAGTTC	
shRNA3 (KD3)	CAGACCTTCCGCAAGATCATTC	GAATGATCTTGCGGAAGGTCTG	

### Viral vector production

A day before transfection, 12 × 10^6^ HEK293T cells were seeded in DMEM supplemented with 10% FBS, 1% penicillin/streptomycin, and 2 Mm L-glutamine. Using the calcium phosphate precipitation method, transfection of HEK293T cells were done with lentivector containing CARs sequence (27 μg/plate) and three packaging plasmids including pMDLg/pRRE pMD. G, pRSV-Rev (9,6,9 μg/plate respectively) in 15 cm tissue culture plate pre-treated with Poly-L-Lysine solution (Sigma, St. Louis, MO) [11]. After 6 h, medium was discarded and replaced with fresh DMEM supplemented with 10% FBS. Virus-containing media (VCM) was then harvested for three times with 12 h intervals and pooled. To remove cell debris, VCM was filtered through 0.45 μm membrane filters, and concentrated by ultracentrifugation at 26,000 rpm for 2 h at 4 °C (Optima LE-80 K Ultracentrifuge, Beckman Coulter, Indianapolis, IN). The viruses were suspended in complete DMEM media (800 μL to each pellet obtained from 50 ml VCM), aliquoted and stored at − 80 °C. Titers of concentrated VCM were determined by a limiting dilution method using flow cytometry on peripheral human T cells which had been activated with anti-CD3/CD28 coated beads.

### Primary T cell isolation, activation, and transduction

Buffy coats or fresh whole blood from healthy donors were purchased from the Iranian Blood Transfusion Organization (IBTO) and handled with necessary safety procedures and ethical requirements. Human peripheral blood mononuclear cells (PBMCs) were isolated by Ficoll–Paque gradient centrifugation. T cells were negatively selected from the PBMCs using immunomagnetic beads (pan T-cell isolation kit II, human, Miltenyi Biotec, Bergisch Gladbach, Germany). Purity of isolated cells was assayed using APC conjugated anti-human CD3 (BioLegend, San Diego, CA). T cells were then activated by anti-CD3/CD28 antibody-coated beads (Cell/Bead ratio: 1/3) (Life Technologies, Grand Island, NY). After 3 days, the anti CD3/CD28 beads were removed and activated T cells were transduced by appropriate amount of concentrated lentiviral supernatants (at multiplicity of infection [MOI] of 7) supplemented with 0.8 μL of polybrene (Santa Cruz Biotechnology, Santa Cruz, CA), then plate was spinofected at 2100 rpm at 32 °C for 1 h, followed by incubation at 37 °C. Six hours later, 1 ml of fresh media containing 100 IU IL-2 (Miltenyi Biotec) was added to each well. After 4 days, CAR expressing cells were detected using goat anti-human IgG F (ab’)2 Biotin (BioRad, Hercules, CA)- Streptavidin APC (BioRad).

### Knockdown experiments

A2aR expression levels were measured by flow cytometry using human Adenosine A2aR Alexa Fluor® 647-conjugated antibody (R&D Systems) according to manufacturer’s instructions. Results were demonstrated as mean fluorescence intensity (MFI) and the percentage of A2aR positive cells. The MFI/percentage of A2aR positive cells was determined both in T cell before activation, activated/un-transduced T cell (Un-T), and activated/transduced T cells (i.e. Mock-transduced and CAR T cells). Percentage of A2aR positive Mock T (T cells that transduced with empty vector) and CAR T cells was also measured after co-incubation with target cells.

Flow cytometric analyses of A2aR knockdown were performed in MSLN-CAR T cell and A2aR.KD1, KD2, and KD3.MSLN-CAR T cell for 6 donors.

### Proliferation assay and cytokine measurement of MSLN-CAR T cells after antigen stimulation

Hela cells were treated with 25 μg/ml mitomycin C (Sigma) for 1 h at 37 °C followed by washing [12]. To track cellproliferation, CAR T, Un-T, and Mock T cells were labeled with PKH26 red fluorescent cell linker (Sigma). The PHK26-labeled cells (2 × 10^5^/well) were co-cultured at a 1:1 ratio with tumor target cells or cultured without target cells as a control of auto proliferation in 48-well plates for 72 h in the presence or absence of 1 μM NECA, and also in the presence or absence of 1 μM SCH58261 in the wells containing MSLN-CAR T cells. For cytokine analysis, supernatant was harvested 24 h after plating and stored at − 80 °C until measurement by the enzyme-linked immunosorbent assay (ELISA). After 72 h, the cell mixture was stained with anti-CD3-APC (BioLegend) to distinguish T cells from target cells. PKH26 dilution in CD3-immunopositive cells was used as a measure of proliferation.

### In vitro cytotoxicity assays

HeLa cells were labeled with PKH26 dye, to distinguish the target cells from T cells. 3 × 10^4^ labeled HeLa cells were co-incubated at 1:1, 1:5, 1:10, and 1:20 ratios with CAR T, Un-T, and Mock T cells for 18 h. Also, 1 μM of the A2aR antagonist SCH58261(Abcam, Cambridge, MA) in the presence and absence of 1 μM 5′-(*N*-ethylcarboxamido) adenosine (NECA- Adenosine receptor agonist) (Abcam). After 18 h of co-incubation, cells were stained with 7-AAD (Miltenyi Biotec) as a viability dye to exclude dead cells and analyzed by flow cytometry. PKH26 positive /7AAD positive population demonstrated dead tumor cells. To calculate the percentage of dead tumor cells, the percentage of spontaneous lysis of target cells which were incubated in the absence of effector cells, were subtracted from the percentage of target cells that were incubated with effector cells. FlowJo (v7.6.1) software was used for data analysis.

### Flow cytometric analysis

Acquisition and analysis of all samples were performed on a BD FACSCalibur (BD Biosciences, San Jose, California) with FlowJo software (v7.6.1). All assays were done in triplicate and repeated three times.

### Statistical analysis

Statistical analyses were performed using ANOVA followed by Tukey’s post hoc comparisons tests to show the difference among treatment groups. Differences were considered statistically significant when *P* < 0.05. Statistical analyses were performed with Prism 7 software.

## Results

### Generation of MSLN-CAR T cell and A2aR targeted MSLN-CAR T cell using lentiviral gene transfer

**Fig. 1:**
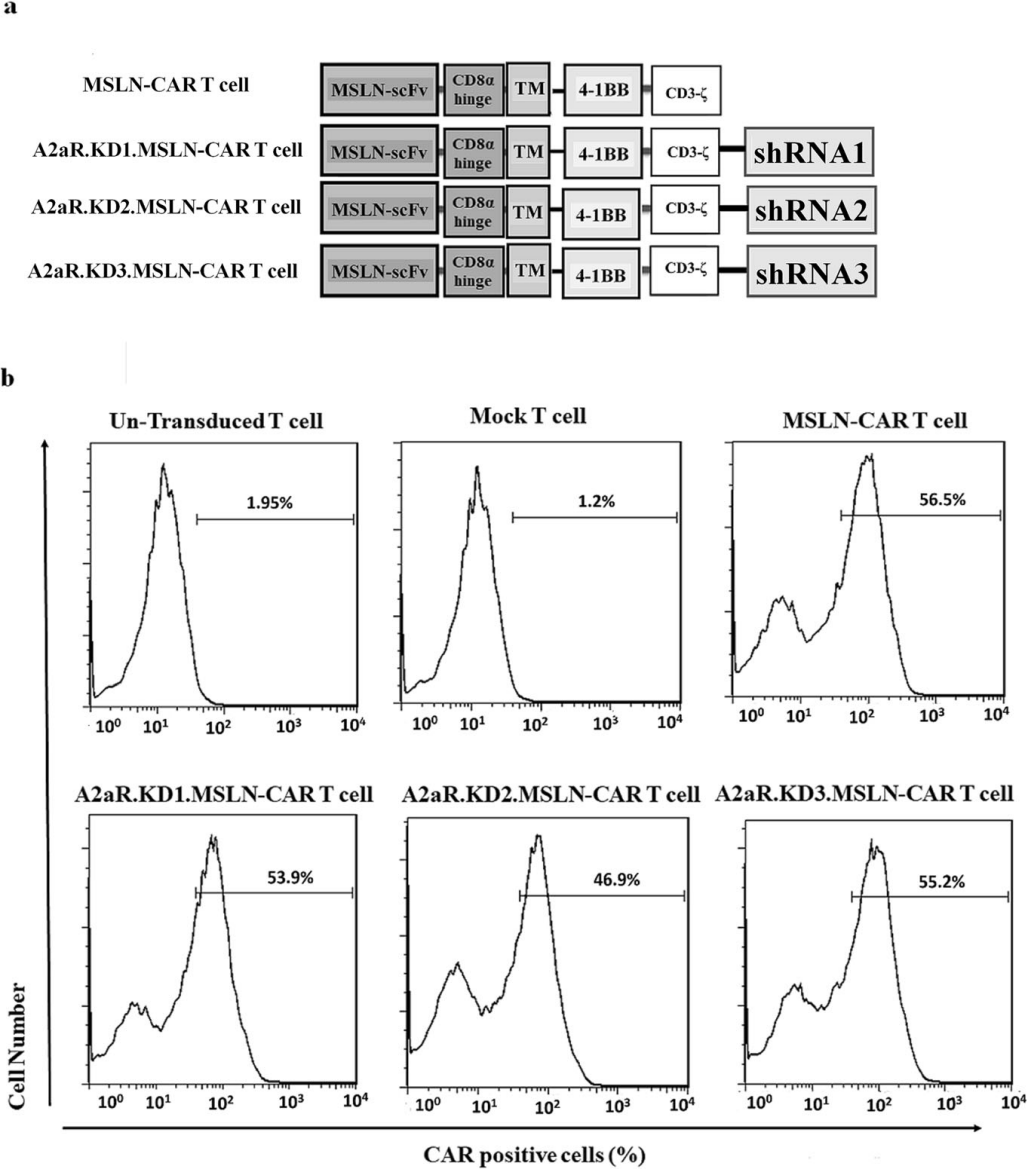
Design and generation of MSLN-CAR T cells without and with A2aR knockdown. **a** Schematic representation of four types of second generation of CAR constructs. MSLN-CAR includes anti-mesothelin (MSLN) scFv, human CD8α-derived hinge, 4-1BB derived transmembrane (TM) domain and two intracellular domains including 4-1BB and CD3ζ. Three A2aR-KD1, 2 and 3-MSLN-CARs included all domains of MSLN-CAR plus an anti A2aR shRNA sequence after CD3ζ sequence. shRNA sequences were designed to target different segments of A2aR gene. **b** Flowcytometry histograms show the percentage of CAR expressing cells 4 days after transduction with the indicated MSLN-CAR-encoding lentiviral vectors at a MOI of ~ 7. The percentage of CAR positive cells is shown. MSLN-CAR: fully human anti mesothelin CAR, A2aR-KD-MSLN-CARs: A2aR-knocked down anti mesothelin-CAR; MOI: multiplicity of infection

As alluded to earlier, in this study we aimed to combine the effects of mesothelin-targeting with diminished expression of A2aR adenosine receptors in CAR T cells. As described in materials and methods, four types of MSLN- CAR T cells were generated and analyzed: MSLN-CAR T cell; i.e. primary human T cells that were transduced with third generation lentiviral particles containing fully human anti mesothelin scFv, a CD8a hinge domain, human 4-1BB TM region, and an intracellular signaling domain containing 4-1BB and CD3ζ domains (Fig. 1a); and three groups of anti-A2aR shRNA-containing CAR T cells were also referred as A2aR.KD1-MSLN CAR T cells, A2aR.KD2-MSLN CAR T cells, and A2aR.KD3-MSLN CAR T cells (Fig. 1a). The other two T cell groups were also used as controls: un-transduced T cells (Un-T), i.e. T cells that passed all CAR T cell production procedures but were not transduced and Mock T cells; i.e. T cells that were transduced by an empty vector. The percentage of CAR expressing cells in all four types CAR T cells was similar, around 50% (Fig. 1b).

### Targeting the A2aR with shRNA leads to efficient A2aR knockdown in the MSLN-CAR T cell

**Fig. 2:**
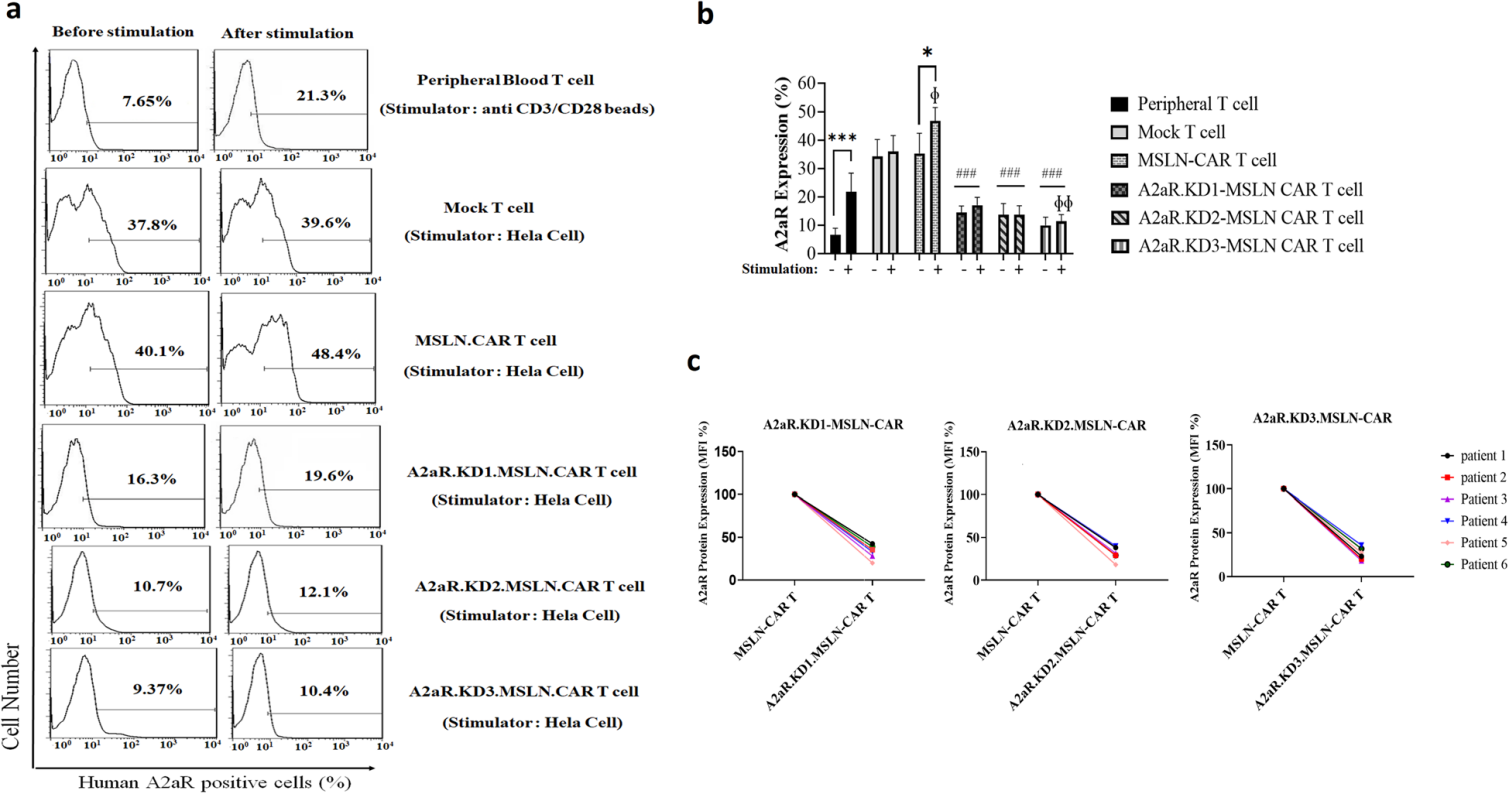
Efficient A2aR knockdown in MSLN-CAR T cells by anti-A2aR shRNA. **a** Human peripheral T cells, Mock T, as well as 4 different groups of CAR T cells from 6 healthy donors were analyzed to determine the percentage of A2aR positive cells. All cells were evaluated before and after stimulation with indicated stimulators. Histogram plots represent percentage of A2aR positive T cell, Mock T cell and CAR T cells from one donor. **b** Bar graph represents the average value for A2aR positive peripheral T cells, Mock T cells and CAR T cells from all 6 donors with or without stimulation. **c** Quantification of mean fluorescence intensity (MFI) demonstrates a reduction in A2aR expression in A2aR.KD1, KD2, and KD3.MSLN-CAR T cells from 6 donors (MFI for MSLN-CAR T cell was set as 100%). * Intragroup comparison of A2aR positive cells before and after stimulation. # Intergroup Comparison of the average levels of the percentage of A2aR positive cells in MSLN-CAR T cell groups with A2aR.KD1, KD2 and KD3.MSLN-CAR T cell groups. ɸ Intergroup Comparison of the percentage of A2aR positive cells in stimulated T cells with stimulated MSLN-CAR T cell groups. *P* < 0.05 were considered as statistically significant calculated with one-way ANOVA with Tukeyʼs post-test for multiple comparisons. MSLN-CAR: fully human anti mesothelin CAR, A2aR-KD-MSLN-CARs: A2aR-knocked down anti mesothelin-CAR VCM: viruses containing media; MFI: mean fluorescence intensity. (*P < 0.05, ^ɸ^*P* < 0.05, ^ɸ ɸ^*P* < 0.01, ^***^P < 0.001, ^###^*P* < 0.001)

To assess the effectiveness of shRNA-mediated knockdown of A2aR, cell surface expression of the receptors was examined in 6 different donors. Flow cytometry with an A2aR-specific antibody showed significant reduction in the percentage of A2aR expressing cells in KD1, KD2, and KD 3 MSLN-CAR T cells compared with MSLN-CAR T cells without shRNA (Fig. 2a). Average A2aR expression in primary T cells, activated Un-T cells, and four types of MSLN-CAR T cell from 6 donors is shown in Fig. 2b. We also found significant reduction in A2aR MFI in A2aR.KDs.MSLN-CAR T cells compared to MSLN-CAR T cells without shRNA (Fig. 2c). Furthermore, we measured A2aR expression on peripheral blood T cells before and after activation with anti CD3/CD28 beads using flowcytometry and also Mock T cell (Fig. 2a and b). The results showed low levels of A2aR expression in un-activated primary T cells in all donors; however, the levels were significantly enhanced following activation with anti CD3/CD28 beads. The percentage of A2aR positive cells showed a significant increase in T and MSLN-CAR T cells after stimulation with anti CD3/CD28 or target cells. There was also a significant increasing of A2aR expression in MSLN-CAR T and Mock T cell even before co-incubation with tumor cells compared to activated T cell (Fig. 2b). However, there was no significant increase in the number of A2aR positive cells in the A2aR.KD1, KD2 and KD3.MSLN-CAR T cell groups. When compared between groups, the percentage of A2aR positive cells was significantly lower in all three A2aR.KD.MSLN-CAR T groups compared with MSLN-CAR T cells (Fig. 2b). The best and weakest efficacy of knockdown belonged to A2aR-KD3 and A2aR-KD1 MSLN-CAR T cells respectively (Fig. 2b and c), but not as significantly (Fig. 2b). However, the A2aR.KD1 and KD3.MSLN were selected to analyze.

### A2aR-targeting enhances proliferation potency of MSLN-CAR T cell

**Fig. 3:**
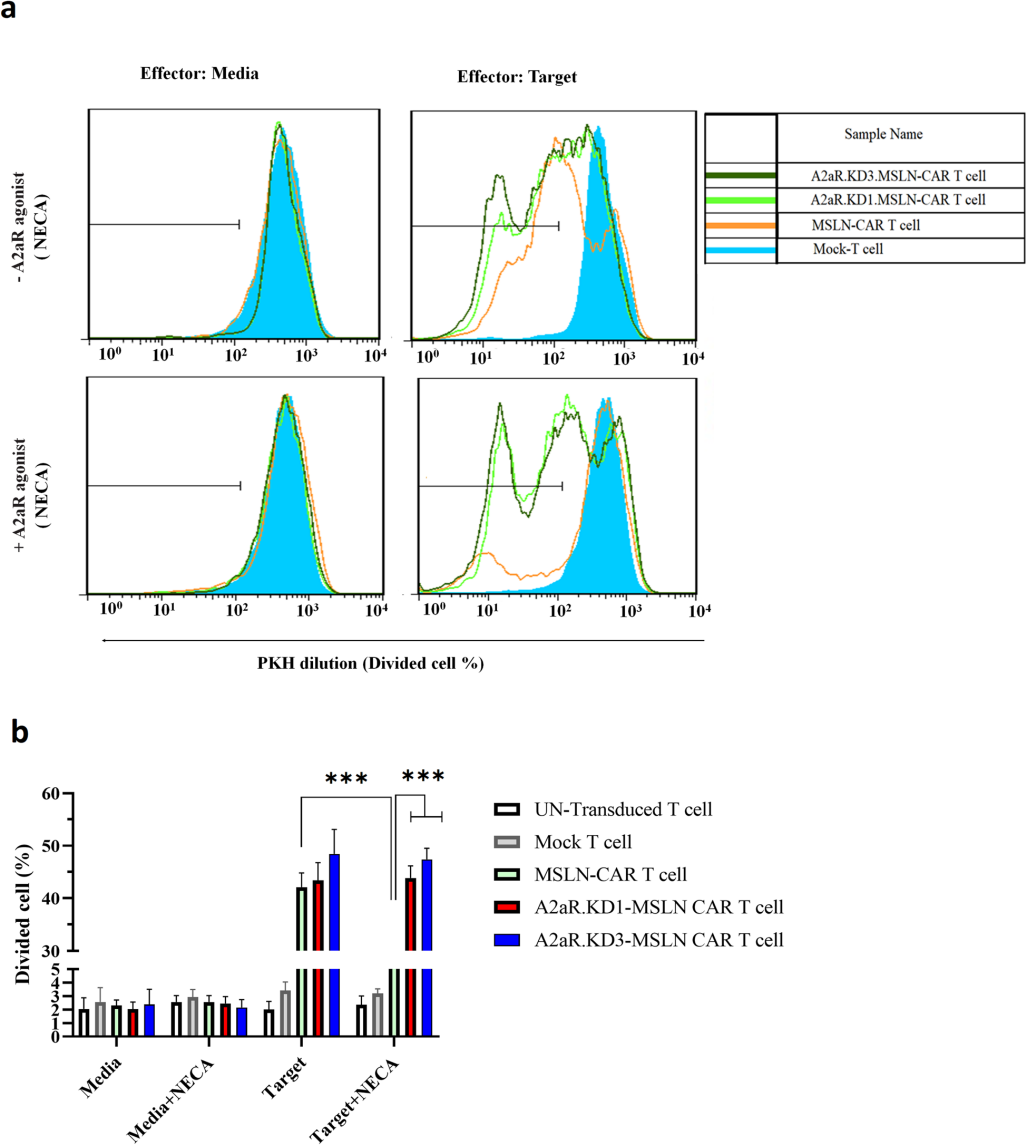
A2aR-knockdown enhances proliferation potency of MSLN-CAR T cell. **a, b** 2 × 10^5^ effector cells including PKH26-Labeled MSLN-CAR T, A2aR.KD1-MSLN.CAR T, A2aR.KD3-MSLN.CAR T, and also Mock T and Un-T as control cells were co-incubated with mitomycin C treated-Hela cell as mesothelin positive target cell at a 1:1 ratio for 72 h in the absence and presence of 1 μM NECA. Anti-CD3 staining was used to distinguish T cells from target cells. PKH dilution was used as a measure of cell proliferation. **a** Histograms display the percentage of divided effector cells. **b** Bar graphs show the average percent of proliferated effector cells in different conditions. Data are presented as mean ± SD from a representative experiment (*n* = 3). MSLN-CAR: fully human anti mesothelin CAR; A2aR-KD-MSLN-CARs: A2aR-knocked down anti mesothelin-CAR; NECA: 5′-(*N*ethylcarboxamido) adenosine; SD: standard deviation. (***P < 0.001)

To investigate the functionality of generated CAR T cells as well as the effects of reduced A2aR expression on their behavior, this study examined the proliferative response of these cells when exposed to mesothelin-expressing HeLa cells, in the presence or absence of adenosine receptor signaling. To this end, all T cell groups were labeled with PKH26 dye, and then co-incubated with equal numbers of mitomycin-treated HeLa cells for 3 days with or without 1 μM of NECA (Fig. 3a and b).

As expected, proliferation levels in all groups of T cells were minimal when exposed to culture medium alone or culture medium containing NECA (Fig. 3b, left columns), as well as, Nalm-6 cell (as mesothelin-negative cell line) (data not published).

When exposed to HeLa cells, control T cells (Un-T and Mock T) did not show any significant response, and proliferation levels were basically the same as the background levels. However, MSLN-CAR T cells as well as A2aR.KD1 and KD3 MSLN-CAR T cells showed a substantial response when exposed to HeLa cells, with the percentage of PKH26 low cells reaching up to 40–50% of the entire cell population (Fig. 3b). To investigate CAR T cells behavior in the presence of active adenosine receptor signaling, a condition which occurs in the adenosine-rich TME, CAR T cells were co-cultured with HeLa cells in the presence of 1 μM of NECA. Presence of NECA significantly reduced the proliferative response of MSLN-CAR T cells, a finding which was consistent with previous reports of adenosine-mediated T cell suppression [13]. Interestingly, A2aR KD1 and KD3 CAR T cells were highly resistant to this suppressive effect, demonstrating significantly higher proliferation levels compared to MSLN CAR T cells. Indeed, percentage of PKH26 low T cells in A2aR.KD1 and KD3 groups were almost the same as in cells exposed to HeLa cells in the absence of NECA..

### A2aR knockdown protects CAR T cell cytotoxic function from negative effects of adenosine signaling

We next analyzed the effect of A2aR knockdown on cytotoxic capacity of MSLN-CAR T cells in the presence or absence of adenosine signaling. Experiments were performed at 1:1, 5:1, 10:1, and 20:1 effector: target cell ratios. The highest level of cytotoxicity was observed at a 20:1 ratio. As expected, all types of MSLN-CAR T cells revealed significantly higher cytotoxic activity compared to Mock T and Un-T even at the lowest target: effector ratios. Also, in exposure to Nalm-6 cell, no significant cytotoxicity function of CAR T cells was seen (data not published).

**Fig. 4:**
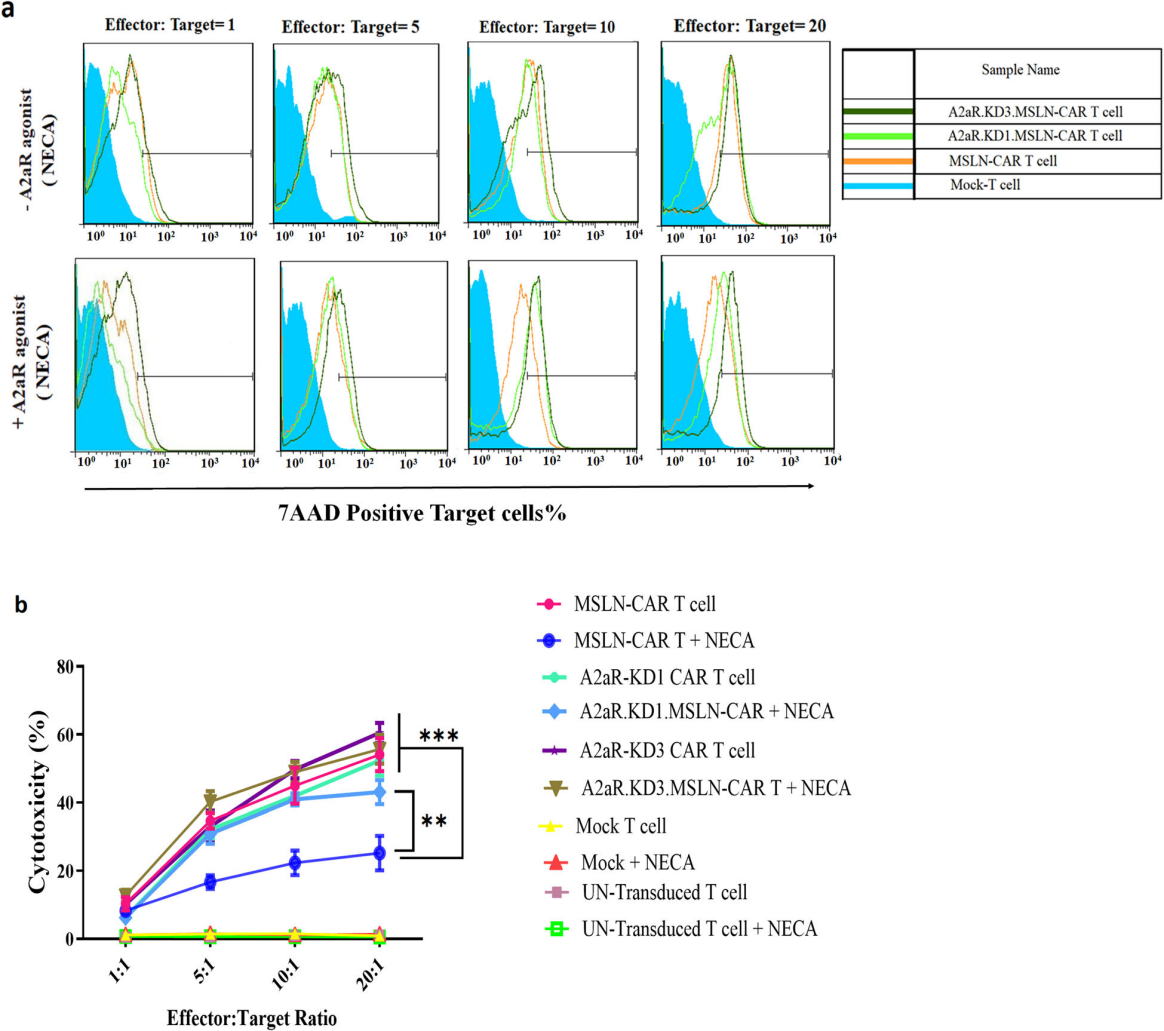
A2aR knockdown prevents inhibition of MSLN-CAR T cell cytotoxicity in the presence of A2aR agonist. **a** Representative flowcytometry histograms show the cytotoxic capacity of MSLN-CAR, A2aR.KD1 and KD3.MSLN-CAR T cell and two controls (Un-T cells and Mock T cells from one donor). 5 × 10^4^ effector cells were mixed with PKH26-labeled HeLa cells as the target at 4 ratios (effector: target) including 1:1, 5:1, 10:1 and 20:1 in the absence or presence of 1 μM NECA A2aR agonist. **b** Line plots display average percent of dead target cells in three independent experiments. MSLN-CAR: fully human anti mesothelin CAR; A2aR-KD-MSLN-CARs: A2aR-knocked down anti mesothelin-CAR; NECA: 5′-(*N*ethylcarboxamido) adenosine; SD: standard deviation. (**P < 0.01, ***P < 0.001)

Different MSLN-CAR T cells were not significantly different in their cytotoxic function in the absence of NECA. However, in the presence of NECA, the killing function of MSLN-CAR T cell was significantly reduced. Interestingly, A2aR.KD1 and KD3-MSLN CAR T cells did not show any significant reduction in cytotoxic function in the presence of NECA (Fig. 4a and b).

### MSLN-CAR T cell cytokine production is enhanced by A2aR knockdown

**Fig. 5:**
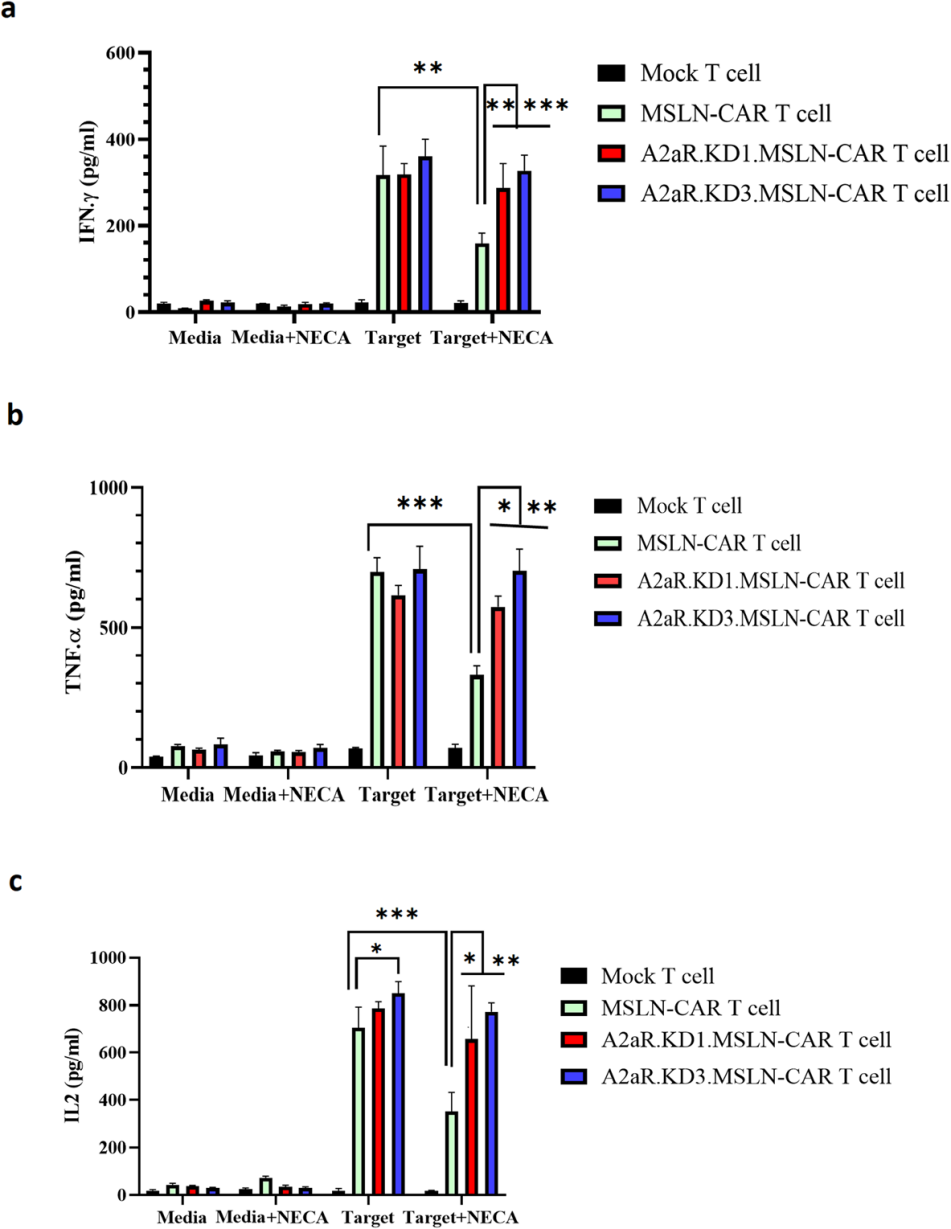
A2aR knockdown provides sustained cytokine secretion by MSLN-CAR T cells in the presence of an A2aR agonist. Mock, MSLN-CAR and A2aR.KD1 and KD3.MSLN-CAR T cells were co-incubated with Hela cells in a 1:1 ratio or incubated in media, in the absence or presence of 1 μM NECA. The supernatant was harvested after 24 h and IFN-γ **(a)**, TNF-α (**b)** and IL-2 (**c)** concentrations were measured by ELISA. Data are shown as mean ± SD. The results are representative of three independent experiments. *P < 0.05 by one-way ANOVA with Tukeyʼs post-test for multiple comparisons. IL: interleukin; MSLN-CAR: fully human anti mesothelin CAR; A2aR-KD-MSLN-CARs: A2aR-knocked down anti mesothelin-CAR; NECA: 5′-(*N*ethylcarboxamido) adenosine; SD: standard deviation. (*P < 0.05, **P < 0.01, ***P < 0.001)

The cytokine production was assessed for different groups of MSLN-CAR T cells. ELISAs were performed for IFN-γ, IL-2 and TNF-α, three key cytokines in cell therapy. Cytokine levels were measured and compared for each group in the presence or absence of NECA (within-group comparison), and also compared cytokine levels between MSLN-CAR T cells and A2aR.KD1 and KD3 MSLN-CAR T cells (Intergroup comparison) (Fig. 5).

Consistent with our previous results, exposure of cells to the culture medium alone or culture medium containing NECA led to minimal cytokine production (Fig. 5a, b, and c left side columns). Exposure of CAR T cells to HeLa cells led to significant induction of IFN-γ (Fig. 5a), TNF-α (Fig. 5b) and IL-2 (Fig. 5c) by all MSLN-CAR T cells, while the levels were minimal in control Un-T and Mock T cells. There was no significant difference in IFN-γ and TNF-α levels between three groups of CAR T cells (Fig. 5a and b). That said, IL-2 levels were higher in A2aR.KD3-MSLN CAR T cells compared to MSLN-CAR T cells.

We next examined cytokine levels in the supernatants of CAR T cells exposed to HeLa cells in the presence of 1 μM of NECA. NECA treatment led to diminished production of IFN-γ, TNF-α and IL-2 by MSLN-CAR T cells compared with MSLN-CAR T cells in the absence of adenosine agonists (Fig. 5a, b, and c). Interestingly, A2aR.KD1 and KD3 MSLN-CAR T cells maintained their robust cytokine response even in the presence of NECA (Fig. 5a, b, and c).

### shRNA targeting of A2aR compared to with A2aR antagonist exhibits more potent effect on MSLN-CAR T cell functions

**Fig. 6:**
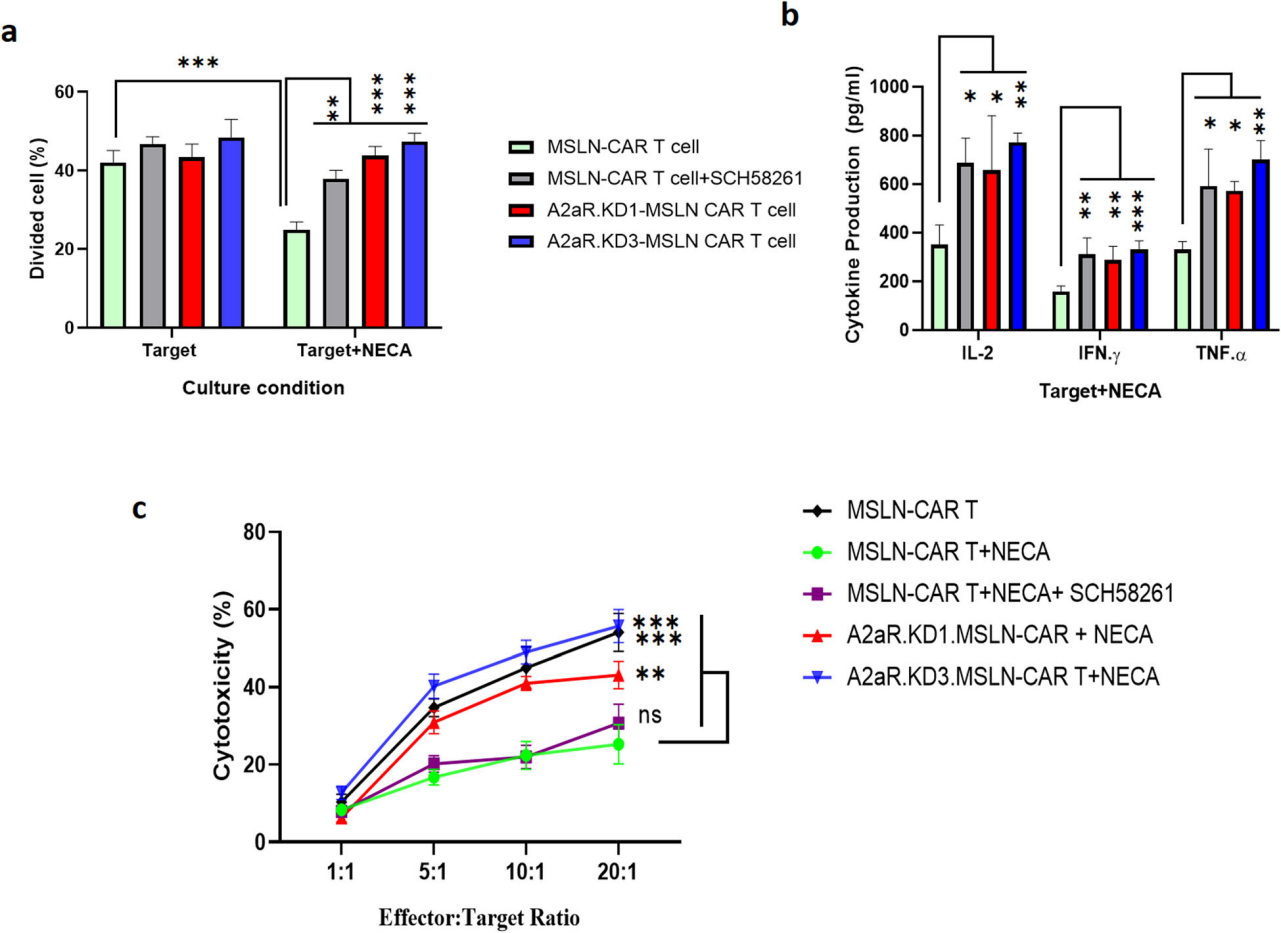
A2aR knockdown outperforms an A2aR antagonist (SCH58261) on MSLN-CAR T cell functions in the presence of an A2aR agonist. Functional analysis of MSLN-CAR T cell including **a** proliferation, **b** cytokine production (IL-2, IFN-γ and TNF-α), and **c** cytotoxicity were performed in the presence of 1 μM A2aR antagonist (SCH58261) and 1 μM NECA. Data are representative of three independent experiments and are shown as mean ± SD. *P* < 0.05 were considered statistically significant. IL: interleukin; MSLN-CAR: fully human anti mesothelin CAR; NECA: 5′-(Nethylcarboxamido) adenosine; ns: Non-significant; SD: standard deviation. (**P < 0.01, ***P < 0.001)

In this study, we decided to compare the efficacy of A2aR shRNA-knockdown with A2aR antagonist (SCH58261) on the MSLN-CAR T cell function (Fig. 6). To this end, all three tested functions (proliferation, cytokine production and cytotoxicity) of MSLN-CAR T cells were done in the presence of 1 μM SCH58261 and 1 μM NECA. Findings revealed a significant positive effect for SCH58261 on MSLN-CAR T cell proliferation (Fig. 6a). Again, SCH58261 significantly enhanced cytokine production by MSLN-CAR T cells compared to cells that had not been treated with the antagonist. The level of up-regulation in cytokine production was similar to A2aR.KD1 and KD3-MSLN CAR T cell groups (Fig. 6b). Despite the effect on proliferation and cytokine production, and unlike shRNA knockdowns, SCH58261 treatment did not show any notable effects on the cytotoxic function on MSLN-CAR T cells (Fig. 6c).

## Discussion

Targeting components of adenosinergic pathway including ectonucleotidases (CD73, CD39) and adenosine receptors has been investigated in cancer-immunotherapy [14–16]. Blocking the effects of adenosine on T cells has been achieved by pharmacological agents, antibodies and molecular methods [17–22]. In the current study, the effects of A2aR gene knockdown and pharmacologic inhibition on MSLN-specific CAR T cells were investigated. We generated MSLN-specific CAR T cells which expressed anti-A2aR shRNAs to knockdown the receptor and an A2aR-specific antagonist to pharmacologically block its function. To simulate TME, we evaluated the function of MSLN-CAR T cells in the presence of adenosine analog, NECA. As expected, adenosine signaling suppressed various aspects of MSLN-CAR T cell function. Targeting A2aR expression by shRNAs led to enhanced proliferation, cytokine production and cytotoxic functions of MSLN-CAR T cells. A2aR pharmacological antagonist also showed similar effects on proliferation and cytokine production, but not cytotoxic ability of the cells.

Numerous studies have shown that adenosine signaling through A2aR negatively regulates T cell function [4, 20, 23] A2aR activation by adenosine has been shown to diminish IL2-dependent proliferation of T cells, induce T-cell anergy and promote the generation of regulatory T cells [24, 25]. These effects are likely mediated by suppression of TCR-mediated signaling through JunB/AP-1, ZAP-70 and ERK1/2 [26, 27]. Adenosine signaling also attenuates T cell cytotoxicity and cytokine production [8]. Enhanced adenosine signaling on T cells could be a consequence of two independent phenomena; increased levels of extracellular adenosine, caused by hypoxic conditions and altered cellular metabolic activity, and increased expression of the receptor as an immune checkpoint molecule on T cells due to activation of the cells following antigen recognition [13, 20, 28]. It is less clear that ex vivo manipulation of T cells used in cancer immunotherapy can influence the expression of A2aR and other inhibitory receptors prior to their administration. In our study, we observed a significant increase in the levels of A2aR on T cells following activation with anti CD3/CD28 (first stimulation). This initial activation is required for efficient transduction during CAR T cell production. Moreover, A2aR-expression was further up-regulated after transduction with lentiviral vectors and co-incubation with target cells. Considering its inhibitory effects, A2aR up regulation might lead to the suppression of CAR T cells even before their exposure to target cells.

Consistent with previous findings with regard to A2aR checkpoint activity [29, 30], A2aR targeting by shRNAs expressed from the CAR construct improved the proliferative response as well as cytotoxic activity of MSLN-CAR T cells in the presence of NECA. This was associated with enhanced production of TNF-α and IFN-γ, cytokines which are indicative of differentiation towards a Th1 [31]. phenotype. IFN-γ influences cytotoxicity of T cells and TNF-α can augment TCR-dependent activation including IL-2 signaling, cell proliferation and cytokine production [32–34]. Pharmacological inhibition of A2aR also reversed the NECA-induced suppression in T cell proliferation and cytokine production. However, it had no effect on decreased cytotoxic function, indicating that molecular targeting of A2aR might be superior to pharmacological inhibition in augmenting CAR T cell activity. Moreover, stable expression of a shRNA sequence ensures constant downregulation of A2aR receptor after infiltration of T cells to the TME, whereas the concentrations of a systemically-administered inhibitor might not reach sufficient levels in tumor tissue.

Another aspect of A2aR signaling which is relevant to cancer immunotherapy is the role of this receptor in promoting the differentiation of regulatory T cells [35]. Factors which might influence the differentiation of Tregs are particularly important in the outcome of tumor immunotherapy, as these cells affect all aspects of anti-tumor response including monocyte/macrophage differentiation, antigen presentation and CD4/CD8 T cell activity. Adenosine and its analogs enhance the production of CD25+ FoxP3+ Tregs and augment their immunoregulatory activity, effects which are mostly mediated by A2aR [25]. Whether CAR T cells which express 4-1BB intracellular domains are sensitive to Treg-promoting signals of A2aR is not clearly known [36]. In this study, we did not evaluate Treg markers before or after exposure to NECA and A2aR knockdown/inhibition. Nonetheless, some of the effects observed after A2aR knockdown (e.g. enhanced proliferation and/or cytotoxic activity) might have been due to suppressed Treg-promoting signal transduction.

## Conclusion

Altogether, the findings of this study demonstrated that A2aR knockdown could have a positive effect on the functionality of MSLN-CAR T cells. Increased expression of A2aR was detected in T cells in different stages of study; i.e.; activation of primary T cells, transduction of activated T cells and exposure to target cells. It seems that CAR T cell production protocols make these cells liable to inhibitory signals. This is an important finding which should be strongly considered in preparation of CAR T cells for clinical applications. Developing new protocols which do not require prior activation might be helpful in generating more potent CAR T cells. A2aR.KD-MSLN CAR T cells seem to have the potential to be used in solid tumor immunotherapy, nonetheless, their activity must be evaluated in vivo and preferably in combination with other immune-check point inhibitors.

## Data Availability

Not applicable.

## Abbreviations

A2aR: Adenosine 2a receptor
ALL: B-cell acute lymphoblastic leukemia
ATP: Adenosine triphosphate
cAMP: Cyclic AMP
CAR T cells: Chimeric antigen receptors
KD: Knockdown
MSLN-CAR T: Fully human anti mesothelin CAR T cells
scFv: Single-chain variable fragments
TME: Tumor microenvironment

## References

[1] Martinez M, Moon EK (2019). CAR T cells for solid tumors: new strategies for finding, infiltrating, and surviving in the tumor microenvironment. Front Immunol.

[2] Chan DA, Giaccia AJ (2007). Hypoxia, gene expression, and metastasis. Cancer Metastasis Rev.

[3] Sitkovsky MV, Kjaergaard J, Lukashev D, Ohta A (2008). Hypoxia-adenosinergic immunosuppression: tumor protection by T regulatory cells and cancerous tissue hypoxia. Clin Cancer Res.

[4] Ohta A, Gorelik E, Prasad SJ, Ronchese F, Lukashev D, Wong MK (2006). A2A adenosine receptor protects tumors from antitumor T cells. Proc Natl Acad Sci.

[5] Bono MR, Fernández D, Flores-Santibáñez F, Rosemblatt M, Sauma D (2015). CD73 and CD39 ectonucleotidases in T cell differentiation: beyond immunosuppression. FEBS Lett.

[6] Klotz K-N (2000). Adenosine receptors and their ligands. Naunyn Schmiedebergs Arch Pharmacol.

[7] Bynoe MS, Viret C (2008). Foxp3+ CD4+ T cell-mediated immunosuppression involves extracellular nucleotide catabolism. Trends Immunol.

[8] Ohta A, Ohta A, Madasu M, Kini R, Subramanian M, Goel N (2009). A2A adenosine receptor may allow expansion of T cells lacking effector functions in extracellular adenosine-rich microenvironments. J Immunol.

[9] Bergan L, Gross JA, Nevin B, Urban N, Scholler N (2007). Development and in vitro validation of anti-mesothelin biobodies that prevent CA125/Mesothelin-dependent cell attachment. Cancer Lett.

[10] Mirzaei HR, Jamali A, Jafarzadeh L, Masoumi E, Alishah K, Fallah Mehrjardi K (2019). Construction and functional characterization of a fully human anti-CD19 chimeric antigen receptor (huCAR)-expressing primary human T cells. J Cell Physiol.

[11] Huang X, Guo H, Kang J, Choi S, Zhou TC, Tammana S (2008). Sleeping beauty transposon-mediated engineering of human primary T cells for therapy of CD19+ lymphoid malignancies. Mol Therapy.

[12] Vincent C, Fournel S, Wijdenes J, Revillard JP (1995). Specific hyporesponsiveness of alloreactive peripheral T cells induced by CD4 antibodies. Eur J Immunol.

[13] Ohta A (2016). A metabolic immune checkpoint: adenosine in tumor microenvironment. Front Immunol.

[14] Clayton A, Al-Taei S, Webber J, Mason MD, Tabi Z (2011). Cancer exosomes express CD39 and CD73, which suppress T cells through adenosine production. J Immunol.

[15] Jadidi-Niaragh F, Atyabi F, Rastegari A, Kheshtchin N, Arab S, Hassannia H (2017). CD73 specific siRNA loaded chitosan lactate nanoparticles potentiate the antitumor effect of a dendritic cell vaccine in 4T1 breast cancer bearing mice. J Control Release.

[16] Arab S, Kheshtchin N, Ajami M, Ashurpoor M, Safvati A, Namdar A (2017). Increased efficacy of a dendritic cell–based therapeutic cancer vaccine with adenosine receptor antagonist and CD73 inhibitor. Tumor Biol.

[17] Young A, Ngiow SF, Barkauskas DS, Sult E, Hay C, Blake SJ (2016). Co-inhibition of CD73 and A2AR adenosine signaling improves anti-tumor immune responses. Cancer Cell.

[18] Young A, Ngiow SF, Madore J, Reinhardt J, Landsberg J, Chitsazan A (2017). Targeting adenosine in BRAF-mutant melanoma reduces tumor growth and metastasis. Cancer Res.

[19] Sek K, Mølck C, Stewart GD, Kats L, Darcy PK, Beavis PA (2018). Targeting adenosine receptor signaling in cancer immunotherapy. Int J Mol Sci.

[20] Beavis PA, Henderson MA, Giuffrida L, Mills JK, Sek K, Cross RS (2017). Targeting the adenosine 2A receptor enhances chimeric antigen receptor T cell efficacy. J Clin Invest.

[21] Cacciari B, Pastorin G, Spalluto G (2003). Medicinal chemistry of A2A adenosine receptor antagonists. Curr Top Med Chem.

[22] Siriwon N, Kim YJ, Siegler EL, Chen X, Rohrs JA, Liu Y (2018). CAR-T cells surface-engineered with drug-encapsulated nanoparticles can ameliorate Intratumoral T cell hypofunction. Cancer Immunol Res.

[23] Hoskin DW, Mader JS, Furlong SJ, Conrad DM, Blay J (2008). Inhibition of T cell and natural killer cell function by adenosine and its contribution to immune evasion by tumor cells. Int J Oncol.

[24] Jenabian M-A, Seddiki N, Yatim A, Carriere M, Hulin A, Younas M (2013). Regulatory T cells negatively affect IL-2 production of effector T cells through CD39/adenosine pathway in HIV infection. PLoS Pathog.

[25] Ohta A, Kini R, Ohta A, Subramanian M, Madasu M, Sitkovsky M (2012). The development and immunosuppressive functions of CD4+ CD25+ FoxP3+ regulatory T cells are under influence of the adenosine-A2A adenosine receptor pathway. Front Immunol.

[26] Linnemann C, Schildberg FA, Schurich A, Diehl L, Hegenbarth SI, Endl E (2009). Adenosine regulates CD8 T-cell priming by inhibition of membrane-proximal T-cell receptor signalling. Immunology.

[27] Sevigny CP, Li L, Awad AS, Huang L, McDuffie M, Linden J (2007). Activation of adenosine 2A receptors attenuates allograft rejection and alloantigen recognition. J Immunol.

[28] Mastelic-Gavillet B, Rodrigo BN, Décombaz L, Wang H, Ercolano G, Ahmed R (2019). Adenosine mediates functional and metabolic suppression of peripheral and tumor-infiltrating CD8+ T cells. J Immunother Cancer.

[29] Leone RD, Sun I-M, Oh M-H, Sun I-H, Wen J, Englert J (2018). Inhibition of the adenosine A2a receptor modulates expression of T cell coinhibitory receptors and improves effector function for enhanced checkpoint blockade and ACT in murine cancer models. Cancer Immunol Immunother.

[30] Leone RD, Emens LA (2018). Targeting adenosine for cancer immunotherapy. J Immunother Cancer.

[31] Romagnani S (2000). T-cell subsets (Th1 versus Th2). Ann Allergy Asthma Immunol.

[32] Aspalter RM, Eibl MM, Wolf HM (2003). Regulation of TCR-mediated T cell activation by TNF-RII. J Leukoc Biol.

[33] Green AM, DiFazio R, Flynn JL (2013). IFN-γ from CD4 T cells is essential for host survival and enhances CD8 T cell function during mycobacterium tuberculosis infection. J Immunol.

[34] Xiao Z, Casey KA, Jameson SC, Curtsinger JM, Mescher MF (2009). Programming for CD8 T cell memory development requires IL-12 or type I IFN. J Immunol.

[35] Ma S-R, Deng W-W, Liu J-F, Mao L, Yu G-T, Bu L-L (2017). Blockade of adenosine A2A receptor enhances CD8+ T cells response and decreases regulatory T cells in head and neck squamous cell carcinoma. Mol Cancer.

[36] Suryadevara CM, Desai R, Farber SH, Choi BD, Swartz AM, Shen SH (2019). Preventing lck activation in CAR T cells confers Treg resistance but requires 4-1BB signaling for them to persist and treat solid tumors in nonlymphodepleted hosts. Clin Cancer Res.

